# Vitamin D Supplementation: Association With Serum Cytokines in Pediatric Hematopoietic Stem Cell Transplantation

**DOI:** 10.3389/fped.2022.913586

**Published:** 2022-07-13

**Authors:** Braden Olsen, Jessica Bodea, Angela Garcia, Kristen Beebe, Courtney Campbell, Carly Schwalbach, Dana Salzberg, Holly Miller, Roberta Adams, Lucia Mirea, Paul Castillo, Biljana Horn, Sandhya Bansal, Thalachallour Mohanakumar, Alexander Ngwube

**Affiliations:** ^1^Creighton School of Medicine, Phoenix, AZ, United States; ^2^St. Jude Children's Research Hospital, Memphis, TN, United States; ^3^Phoenix Children's Hospital, Phoenix, AZ, United States; ^4^Mayo Clinic Arizona, Phoenix, AZ, United States; ^5^UF Health Shands Children's Hospital, Gainesville, FL, United States; ^6^St. Joseph's Hospital & Medical Center, Phoenix, AZ, United States

**Keywords:** vitamin D, hematopoietic cell transplantation, pediatric, cytokine-immunological terms, transplant complications

## Abstract

Vitamin D deficiency is prevalent in pediatric patients presenting for hematopoietic stem cell transplantation (HSCT) and has been linked to poor clinical outcomes. Using the data from a randomized control trial, in this paper we explore the effects of vitamin D supplementation on circulating cytokine levels during pediatric HSCT (www.clinicaltrials.gov as NCT03176849). A total of 41 children, 20 received Stoss therapy and 21 children received standard of care vitamin D supplementation. Levels of 25(OH)D and 20 cytokines were assessed at baseline and day +30. Significantly (*P* < 0.05) higher levels of mostly proinflammatory cytokines, FGF, GCSF, TNFα, IL-2, IL-6, IP10 were detected pre-transplant for patients with low compared to those with normal vitamin D levels. In sex stratified models that compare changes in cytokines between Stoss vs. standard of care, females in the Stoss group show greater changes in mostly pro -inflammatory cytokines- IP-10 (*P* = 0.0047), MIG (*P* = 0.009), and RANTES (*P* = 0.0047), IL-2R (*P* = 0.07) and IL-6(*P* = 0.069). Despite a small sample size, these findings suggest vitamin D deficiency affects the pre-transplant cytokine milieu and higher doses of vitamin D (Stoss therapy) appears to influence proinflammatory cytokine responses in a sex specific manner during pediatric HSCT. Larger clinical trials are warranted to validate these results.

## Introduction

Hematopoietic stem cell transplantation (HSCT) is a critical treatment for a constellation of diseases including lymphoma, leukemia, immune-deficiency illnesses, congenital metabolic defects, hemoglobinopathies, and myelodysplastic and myeloproliferative syndromes (1). However, treatment is aggressive, often requiring “wiping” the immune system, and adverse outcomes are unfortunately common. HSCT treatment can lead to increased risk for infections, systemic inflammation, and graft-versus-host disease (GvHD) (2). GVHD is a condition that occurs when donated hematopoietic stem cells (the graft) see the healthy tissues in the patient's body (the host) as foreign and attack them. During HSCT, the combination of the conditioning regimen and the alloreactive response from donor cells is known to trigger a systemic inflammatory response (3, 4). Cytokines—small proteins released by the immune system—tend to be the main drivers of this proinflammatory response (5).

Evidence from multiple studies indicates that elevated proinflammatory cytokines are linked to adverse events and worse outcomes. In a sample of pediatric patients (*N* = 51) receiving HSCT, DiCarlo et al. (6) report that 2–4 weeks post-HSCT hepatocyte growth factor and interleukin(IL)-6 surged in children that later developed complications such as sinusoidal obstructive syndrome (SOS) and GvHD. Similarly, in a study of post-transplant adverse events in pediatric patients (*N* = 61), Döring et al. (7) report significantly increased levels of IL-6, IL-8, and tumor necrosis factor α (TNF-a) in accompaniment with SOS, and IL-10, sIL2-R, IL-6, and TNF-a with GvHD.

Vitamin D is a key immunomodulating agent that plays important roles in both the innate and adaptive arms of the immune system (8–10). It acts primarily through selective binding to the vitamin D receptor—expressed nearly ubiquitously in immune cells and hematopoietic stem cells— and regulation of gene transcription (11). Vitamin D inhibits B cell proliferation and blocks differentiation and immunoglobulin secretion, as well as suppressing T cell proliferation (12). Vitamin D further inhibits monocyte production of proinflammatory cytokines such as IL-1, IL-6, IL-8, IL-12, TNF-a (12). Collectively, these actions result in decreased production of inflammatory cytokines, and increased production of anti-inflammatory cytokines, thus shifting from a pro-inflammatory toward a tolerogenic state (9, 13).

In a recently published meta-analysis, COVID-19 patients with low (<30 μg/L) serum vitamin D (25-hydroxyvitamin D, i.e., [25(OH)D]) levels had abnormally high serum troponin and peak D-dimer levels, as well as elevated IL-6 and C-reactive protein than those with vitamin D levels ≥30 μg/L. Similarly, patients with low vitamin D status had statistically higher risk of developing severe COVID-19 pneumonia and a higher risk of death (25). In addition, inverse associations between serum vitamin D and proinflammatory cytokines have been documented in numerous chronic inflammatory disorders. In juvenile onset systemic lupus erythematosus, proinflammatory cytokines interferon (IFN)-γ and IL-17 were significantly higher, while 25(OH)D was significantly lower compared to controls (14). In a study looking at immune reconstitution of tuberculosis patients with inflammatory syndromes, Conesa-Botella, et al. reported that patients with lower circulating levels of 25(OH)D were found to have higher baseline levels of TNF-α, IL-6 and IL-8 (15). Furthermore, Gubatan et al. (16) in patients with ulcerative colitis and Kelly et al. (17) in patients with Crohn's disease, both demonstrated an association between higher serum vitamin D and increased IL-10 levels, an anti-inflammatory cytokine.

Despite the established links between vitamin D and cytokines, the relationship between vitamin D and cytokine response during HSCT is not well-documented, particularly in children. Assessing the effects of vitamin D on cytokines in the context of HSCT is necessary to fully evaluate vitamin D supplementation as part of a treatment plan for HSCT patients. Using data collected as part of a randomized trial, here we examined the effects of two different courses of vitamin D supplementation on cytokines induced during HSCT in pediatric patients.

## Methods

### Study Setting, Participant Recruitment, and Vitamin D3 Dose Monitoring

The sample for this study included pediatric patients from a prospective randomized trial (completed in 2020) that evaluated the safety and efficacy of vitamin D supplementation of an intervention (Stoss) therapy compared to a standard of care Vitamin D supplementation regimen for pediatric patients undergoing HSCT (ClinicalTrial.org ID: NCT03176849). The original trial methodology has been described previously (18). Briefly, study subjects were pediatric patients ages 1–21 years, undergoing allogenic HSCT for either malignant or non-malignant indications at Phoenix Children's Hospital and the University of Florida Health Shands Children's Hospital. The present study examined 41 (85%) of the 48 subjects that completed the original randomized trial; one patient was excluded due to missing baseline cytokine values; six patients were excluded due to receiving auto transplant. All subjects received standard supportive care required during allogeneic HSCT.

In the original study, the intervention—Stoss therapy— consisted of a single oral dose of vitamin D administered on the day of admission (prior to onset of conditioning regimen) and followed 1 week later with maintenance therapy. The initial Stoss dose ranged from 100,000 to 600,000 IU and was based on baseline 25(OH)D level and age. Maintenance therapy included 400–600 IU/day for those with pre-transplant vitamin D levels ≥30 μg/L, and 50,000 IU/week for those with pre-transplant levels of <30 μg/L (18).

The standard of care (SoC) dosing was in accordance with the Endocrine Society Guidelines and institutional policy: those at risk of becoming deficient received 400–600 IU/day; those found to be deficient or insufficient were given 50,000 IU/week through 100 days following HSCT. All research procedures were approved by the Institutional Review Boards at both institutions. Written assent / consent to participate was collected from both children and their parents.

### Sample Collection and Diagnostic Testing

Whole blood (~8 ml) was collected by venipuncture at baseline and day +30 post-HSCT. Serum was isolated by centrifugation and stored at −80°C prior to measurement of 25(OH)D performed by immunoassay. The analysis of a 20-cytokine panel was performed on thawed serum samples using a commercially available multiplex immunoassay (ThermoFisher Cytokine Human Panel Kit, BD) according to the manufacturer's protocol. Cytokines examined included *growth factors*: epidermal growth factor (EGF), basic fibroblast growth factor (FGF-basic), granulocyte-colony stimulating factor (G-CSF), hepatocyte growth factor (HGF); *pro-inflammatory classics*: tumor necrosis factor α (TNF-α), interleukin (IL) 1a, IL-1β, IL-1 receptor antagonist (IL-1RA*), type 1*: IL-12, IL-2, IL-2 receptor (IL-2R), *type 2*: IL-3, IL-4, IL-6 and *chemokines*: IL-8, inducible protein 10 (IP-10), monocyte chemotactic protein 1 (MCP-1), monokine induced by IFN-γ (MIG), macrophage inflammatory protein-1β (MIP-1-ß), RANTES and eotaxin. The concentration of each cytokine was quantified by the Bio-Plex 200 System (Bio-Rad Laboratories, Inc.). Vitamin D (25(OH)D) is reported in μg/L; cytokine levels are reported in pg/ml. All experimental assays were performed by research personnel blinded to the vitamin D supplementation status of participants.

### Statistical Analysis

Initial analyses compare characteristics between patients with low vs. normal vitamin D levels. For these analyses, patients were split into groups determined by pre-HSCT vitamin D levels: “normal” is defined as serum vitamin D levels ≥30 μg/L, and “low/insufficient” is serum vitamin D <30 μg/L. Demographic and baseline characteristics were summarized and compared using the Fisher-exact or Wilcoxon rank sum test. Cytokine and vitamin D levels were compared using Fisher-exact or Wilcoxon rank sum tests at two time points: pre-HSCT and HSCT+30.

Longitudinal modeling of vitamin D and cytokine associations was based on intervention. To evaluate the effect of vitamin D on cytokines based on the dosing regimen from the original clinical trial (Stoss vs. SoC), we calculated within-individual changes from pre-HSCT to day HSCT+30 for levels of vitamin D and each cytokine. Using generalized estimating equations with robust variance (to account for non-normal distributions in biomarker data) we modeled associations between slopes of change of vitamin D and each cytokine. Intervention groups were modeled separately to account for effect modification (sample sizes for each model: Stoss [*n*=19], SoC [*n* = 22]). The interaction between sex and change in cytokine levels between baseline and day 30 was assessed to determine whether the association of Stoss vs. SoC on cytokine change was different for females and males.

All statistical tests were two-sided with significance evaluated at the 5% level. Statistical analyses and graphics were performed using the software packages SAS v9.4 (Cary, NC), IBM SPSS V.23 (NY, US) and GraphPad Prism V.6.0 (CA, US).

## Results

### Participant Demographics

Study subjects included 41 children (20 females and 21 males) with a median age of 14 years (interquartile range 7–16); 23 (56%) patients had malignant disorders and 18 (44%) had non-malignant disorders (Supplementary Table 1). Pre-HSCT median serum 25-hydroxy vitamin D was 24 μg/L in this sample. Vitamin D levels at baseline (pre-HSCT) were normal (≥30 μg/L) in 12 (29%) and low/insufficient (<30 μg/L) in 29 (71%) subjects. Neutrophil and platelet engraftment was similar between low vitamin D and normal vitamin D groups (neutrophil engraftment: 16 days (range 15–25.5) vs. 14.5 days (range 13–17), *P* = 0.13 and platelet engraftment: 20 days (range 17–20) vs. 24.5 days (range 18–32), *P* = 0.32, respectively. No statistically significant differences were detected in the distribution of demographic or baseline clinical factors between normal or deficient/insufficient vitamin D groups (Table 1).

**Table 1:** Demographic and characteristics of HSCT patients according to Vitamin D levels at baseline.

**Characteristic**	**All subjects (*N* = 41)**	**Vitamin D level at baseline**		***P*-value**
		**Low <30 μg/L** **(*N* = 29)**	**Normal ≥30 μg/L (*N* = 12)**	
**Age (years)**
Mean (SD)	12 (5.1)	12 (4.9)	9.8 (5.4)	0.20
Median (IQR)	14 (7, 16)	14 (9, 16)	10 (4.8, 14)	
**Sex**, ***N*** **(%)**
Female	20 (49)	16 (55)	4 (33)	0.11
Male	21 (51)	13 (45)	8 (67)	
**Weight (kg)**
Mean (SD)	47 (25)	51 (26)	35 (19)	0.05
Median (IQR)	46 (29, 62)	55 (31, 68)	34 (17, 49)	
**Diagnosis**, ***N*** **(%)**
Malignant	23 (56)	16 (55)	7 (58)	1.0
Non-malignant	18 (44)	13 (45)	5 (42)	
**Vitamin D levels at baseline (μg/L)**
Mean (SD)	25 (12)	19 (6.3)	40 (9)	–
Median (IQR)	24 (17, 31)	18 (13, 25)	39 (32, 44)	

### Comparing Vitamin D and Cytokine Levels by Vitamin D Status at Baseline and +30

**Figure 1:**
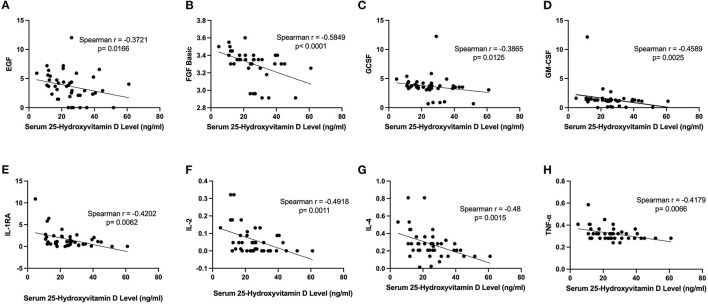
Negative correlation between serum vitamin D levels and inflammatory cytokine profiles. **(A)** Serum vitamin D levels and serum levels of EGF (*r* = −0.3721, *P* = 0.0166). **(B)** Serum vitamin D levels and serum FGF-basic (*r* = −0.5849, *P* < 0.01). **(C)** Serum vitamin D levels and serum GCSF (*r* = −0.3865, *P* = 0.0125). **(D)** Serum vitamin D levels and serum levels of GMCSF (*r* = −0.4589, *P* < 0.01). **(E)** Serum vitamin D levels and serum IL-1RA (*r* = −0.4202, *P* < 0.01). **(F)** Serum vitamin D levels and serum IL-2 (*r* = −0.4918, *P* < 0.01). **(G)** Serum vitamin D levels and serum levels of IL-4 (*r* = −0.48, *P* < 0.01). **(H)** Serum vitamin D levels and serum TNF-α (*r* = −0.4179, *P* < 0.01).

At baseline, higher serum vitamin D levels correlated negatively with decreased serum cytokine FGF, GCSF, GMCSF, IL-1ra, IL-2, IL-4, IL6 and TNF-α, Figure 1. In addition, Table 2 summarizes and compares the distribution of baseline serum cytokine levels by vitamin D status at baseline. Mean levels were significantly higher in subjects with low/insufficient vs. normal Vitamin D across several cytokines: EGF (4.26 vs. 2.57 pg/ml, *P* = 0.02), FGF (3.36 vs. 3.21 pg/ml, *P* = 0.004), IL-2 (0.08 vs. 0.02 pg/ml, *P* = 0.01), IL-4 (0.31 vs. 0.19 pg/ml, *P* = 0.01), and IL-6 (0.41 vs. 0.17 pg/ml, *P* = 0.003). In contrast, the anti-inflammatory cytokine HGF was significantly lower (0.0 vs. 36.0 pg/ml, *P* = 0.03) in patients with low/insufficient compared to those with normal vitamin D levels. All other cytokines measured did not differ between groups (all *P*-values > 0.1; Table 2).

**Table 2:** Association of cytokines and vitamin D levels at baseline.

**Cytokine**	**Baseline vitamin D Mean (SD)**			**P-value**	**Mean difference (95% CI)**
	**All subjects (*N* = 41)**	**<30 μg/L (*N* = 29)**	**≥30 μg/L (*N* = 12)**		
**Growth factors**
EGF	3.76 (2.43)	4.26 (2.44)	2.57 (2.04)	**0.03**	−1.70 (−3.23, −0.16)
FGF	3.31 (0.17)	3.36 (0.14)	3.21 (0.19)	**0.004**	−0.15 (−0.28, −0.02)
GCSF	3.60 (1.76)	3.88 (1.86)	2.93 (1.33)	0.07	−0.96 (−2.02, 0.10)
HGF	10.5 (60)	0 (0)	36.0 (92)	**0.03**	36.0 (−22.4, 94.4)
**Proinflammatory classic**
TNFα	0.32 (0.06)	0.34 (0.07)	0.30 (0.03)	0.06	−0.04 (−0.07, −0.01)
IL1A	0.13 (0.36)	0.15 (0.42)	0.06 (0.12)	0.36	−0.09 (−0.27, 0.08)
IL1β	3.18 (2.68)	3.24 (2.71)	3.03 (2.72)	0.48	−0.21 (−2.14, 1.74)
IL1RA	1.60 (2.06)	1.92 (2.32)	0.84 (0.87)	0.07	−1.07 (−2.08, −0.06)
**Type 1**
IL12	4.14 (5.05)	3.95 (4.81)	4.57 (5.80)	0.98	0.62 (−3.38, 4.61)
IL2	0.06 (0.08)	0.08 (0.09)	0.02 (0.04)	**0.01**	−0.06 (−0.10, −0.02)
IL2R	6.41 (34)	8.74 (41)	0.78 (2.32)	0.33	−7.96 (−23.6, 7.63)
**Type 2**
IL3	0.53 (0.31)	0.52 (0.26)	0.54 (0.43)	0.82	0.02 (−0.26, 0.31)
IL4	0.28 (0.17)	0.31 (0.19)	0.19 (0.07)	**0.01**	−0.12 (-0.21, −0.04)
IL6	0.34 (0.88)	0.41 (0.98)	0.17 (0.56)	**0.003**	−0.24 (−0.74, 0.25)
**Chemokines**
IL8	3.55 (9.06)	3.63 (9.01)	3.35 (9.58)	0.66	−0.28 (−7.04, 6.47)
IP10	3.72 (4.10)	4.47 (4.45)	1.89 (2.36)	0.07	−2.58 (−4.75, −0.41)
MCP1	102 (303)	126 (357)	43.6 (66)	0.60	−82.8 (−223, 58)
MIG	9.08 (48)	12.6 (56)	0.58 (0.98)	0.31	−12.0 (−33.4, 9.41)
MIP1β	3.94 (5.46)	3.94 (5.77)	3.95 (4.87)	0.37	−0.01 (−3.64, 3.65)
RANTES	137 (37)	131 (40)	151 (26)	0.10	20.1 (−1.24, 41.52)
EOTAXIN	6.79 (3.99)	7.58 (4.25)	5.14 (2.76)	0.12	−2.34 (−4.63, −0.05)

*Bold values indicate the SD: standard deviation*.

There were no significant differences observed in day +30 mean cytokine levels between normal and low/insufficient vitamin D status at day +30 (Supplementary Table 2).

### Comparing Levels of Vitamin D and Cytokines by Intervention Type

At Day +30, vitamin D levels were significantly higher in the Stoss regimen (42.3 ± 12 μg/L) compared to standard of care (35.8 ± 14.0 μg/L) (*P* = 0.05), see Figure 1. In the intervention (Stoss) group, subjects with inadequate vitamin D levels dropped from 64% to 14% compared to subjects in control (SoC) group that dropped from 77% to 35%.

As expected by design, baseline (pre-HSCT) vitamin D and cytokine levels did not vary between patients randomized to Stoss or standard of care vitamin D supplementation groups (Supplementary Table 3). Further, no significant differences were detected in mean cytokine concentrations at day +30 between Stoss and standard of care treatment groups (Supplementary Table 3).

### Change in Cytokine Concentrations Based on Intervention Type

Last, we performed exploratory analyses to evaluate whether there were differences in slope of change in cytokine levels between baseline and day +30 based on intervention type. Overall, there were no differences in change in cytokine concentration based on intervention type, except for RANTES, which decreased among patients in the Stoss group, but increased for those in SoC (−19.8 pg/ml vs. 20.9 pg/ml, *P* = 0.0105).

In sex stratified models that compare changes in cytokines between Stoss vs. SoC, we find that females in the Stoss group show greater changes in mostly pro-inflammatory cytokines- IP-10 (−2.7 vs. 3.3 pg/ml, *P* = 0.0047), MIG (−7 vs.−22.9 pg/ml, *P* = 0.009), and RANTES (−13.3 vs. 29.6 pg/ml, *P* = 0.0047). Additionally, IL-2R (0 vs. 49.9 pg/ml, *P* = 0.07) and IL-6 (−0.5 vs. 1.8 pg/ml, *P* = 0.069), although not statistically significant. In contrast, for males, there was no difference by group (Stoss vs. SoC) in changes across any of the cytokines (Table 3). In addition, conditioning regimen/diagnosis type stratified models that compare changes in cytokines between Stoss vs. SoC, we find that patients that received myeloablative regimen (all malignant disorders) in the Stoss group show greater changes in pro-inflammatory cytokines- HGF (−22.5 vs. 28.1 pg/ml, *P* = 0.045), and RANTES (−33.7 vs. 11.9 pg/ml, *P* = 0.0448). In contrast, for reduced intensity conditioning regimen (all non-malignant disorders), there was no difference by group (Stoss vs. SoC) in changes across any of the cytokines.

**Table 3:** Selected cytokine with significant changes by intervention when stratified by sex.

**Cytokine**	**Cytokines change in females mean (SD)**				**Cytokines change in males mean (SD)**			
	**Total *N* = 20**	**Stoss *N* = 7**	**SoC *N* = 13**	***P*-value**	**Total *N* = 21**	**Stoss *N* = 12**	**SoC *N* = 9**	***P*-value**
IP10	1.1 (6.2)	−2.7 (7.4)	3.3 (4.4)	**0.047**	3.1 (5.8)	4.6 (6.2)	1.1 (5.0)	0.283
MIG	−17.1 (68.7)	−7.1 (9.6)	−22.9 (87.0)	**0.01**	3.6 (8.8)	5.5 (11.1)	1.0 (3.4)	0.099
Rantes	13.8 (54.7)	−13.3 (23.2)	29.6 (62.2)	**0.047**	−10.6 (54.5)	−23.9 (68.5)	7.8 (16.5)	0.173
IL-2R	31.5 (136.7)	0.0 (2.5)	49.9 (172.0)	0.071	8.3 (15.4)	13.9 (17.8)	0.6 (6.4)	0.06
IL-6	1.0 (5.2)	−0.5 (1.4)	1.8 (6.5)	0.069	16.2 (69.9)	27.8 (91.9)	0.3 (0.6)	0.8

*Bold values indicate the SD: standard deviation*.

## Discussion

This study examined the association between vitamin D supplementation and patterns of cytokine profiles during HSCT in pediatric patients. We find that at baseline, children with low vitamin D had significantly higher levels of several pro-inflammatory cytokines analyzed (EGF, FGF, IL-2, IL-4, and IL-6) compared to subjects with normal levels of vitamin D. However, vitamin D supplementation (regardless of intervention type) diminished differences in +30-day (post-HSCT) levels of all cytokines measured. Our findings of baseline differences provide evidence for a similar association between vitamin D and pro-inflammatory immune function in pediatric HSCT patients, as what has been reported in larger studies in adult or mixed age samples (19–25). Multiple studies have reported temporal association between pre-transplant vitamin D deficiency and prognosis of HSCT (26–29), likely due in part to the pro-inflammatory phenotype that accompanies low vitamin D levels. That cytokine levels were comparable between groups following either Stoss or SoC suggests that vitamin D supplementation may be beneficial at least to minimize differences based on pre-transplant vitamin D status. During HSCT, conditioning regimen causes tissue, endothelium, and epithelia damage, resulting in a production of several pro-inflammatory cytokines (3, 4). These molecules propagate the inflamed microenvironment where antigen presenting cells (monocytes and dendritic cells), NK cells, and T lymphocytes sustain the inflammation and maintain activation of lymphocytes (4). In this context, our analysis reveals, some differences by intervention type in females, but not males. In our mostly peri-pubertal females, we found decrease in pro-inflammatory cytokines- IP-10, RANTES and though not statistically significant -IL-2R and IL-6- in the Stoss intervention arm compared to the SoC regimen (Table 3). MIG was more decreased in the SoC arm compared to the Stoss. These proinflammatory cytokines are T cell chemoattractant cytokines (30), and in the allogenic HSCT setting, high levels of these cytokines have been associated with several immune-mediated complications, including GvHD, acute renal injury idiopathic pneumonia syndrome and,graft failure and low overall survival of patients undergoing transplantations (31–35).

In some autoimmune diseases, gender differences have been reported in the immunomodulatory effects of vitamin D3 (36). Correale et al. (37) observed that vitamin D3 induces a stronger inhibition of the production of pro-inflammatory cytokines and a higher increase of anti-inflammatory cytokines in lymphocytes from multiple sclerosis female patients in contrast to those from male patients. The exact reason for this difference is still being explored but it has been reported that vitamin D3 acts in an estrogen-dependent manner in controlling T regulatory cell differentiation (38). Estrogen increases the expression of the nuclear vitamin D receptor gene in CD4+ T cells and decreases the expression of CYP24A1, the cytochrome P450 component of the 25-hydroxyvitamin D(3)-24-hydroxylase enzyme which inactivates vitamin D3 (39). These may explain the molecular mechanism by which sex differences affects vitamins' immune modulation in certain disorders. Hence, our results lead us to hypothesize that higher doses of vitamin D supplementation particularly in females modulates the overexpression of several proinflammatory cytokines that can contribute to vital organ compromise, multisystem dysfunction syndrome and possible death after transplantation. Larger studies should be done to confirm this observation and further investigations into sex-specific molecular mechanisms that regulate the effects of vitamin D on cytokine profile during HSCT may help to identify targets that could mitigate some of the early complications of transplant.

Allogenic HSCT is associated with events which agitate the normal steady state of circulating cytokines, however cytokine changes during transplant are likely to vary substantially according to the regimen, type of transplant and leucocyte recovery (40). Remberger, et al. (41) in a study looking at patients with mainly malignant disorders showed that reduced intensity conditioning regimen was shown to induce increased TNFα levels compared to myeloablative therapy. In contrast, Grimley, et al. (42) observed increased pro-inflammatory cytokines (IL-6, G-CSF, and IL-2Rα) in patients with malignant and non-malignant disorders who received myeloablative conditioning (MAC) compared to non-malignant patients who received reduced intensity conditioning regimen (RIC). In our study we did not see any differences in cytokine profile comparing RIC to MAC or non-malignant disorders to malignant disorders during transplant. However, we observed that higher doses of vitamin D supplementation (Stoss therapy) induced a stronger inhibition of the production of pro-inflammatory cytokines (HGF and RANTES) from malignant patients who all received MAC in contrast to non-malignant patients who all received RIC. Once again, larger studies should be done to confirm this observation with further investigations into conditioning specific molecular mechanisms that regulate the effects of vitamin D on cytokine profile during HSCT.

The effects of vitamin D supplementation on human immunology have now been evaluated by several studies, including large scale randomized controlled trials, with conflicting results. One study reported no effect of vitamin D supplementation on circulating cytokines, such as IL-6 or C reactive protein (43). However, a randomized controlled trial by Schleithoff et al. (44) showed that (2,000 IU/day) of vitamin D3 reduced the inflammatory milieu in congestive heart failure patients. Yegorov et al. (45) in their randomized control study in healthy Mongolian children, also showed that 800 IU of vitamin D daily was associated with elevated IL-6. The placebo group had reduced macrophage inflammatory protein (MIP)-1α and IL-8 at 6 months. These seemingly contradictory findings could be attributed to various confounders and differences among the studies, such as the duration and dosage of vitamin D supplementation, underlying clinical conditions, effects of clinical therapy, and extent of vitamin D deficiency at baseline.

We recognize that this study has several limitations. Cytokine data were imputed, based on optical densities which can be unreliable. As a secondary analysis, sample size calculation was not performed prior to recruitment and data collection, and consequently, statistical power may have been inadequate to detect small differences in blood cytokine concentrations due to high inter-individual variability. In addition, variables such as donor source (cord blood, bone marrow, peripheral blood), donor type (matched vs. mismatch), all of which may impact the baseline and post-transplant cytokine levels, exist but were not included in the statistical model given the small overall sample size and small effect sizes. However, statistical analysis comparing changes in cytokine concentrations intra-individually in relation to vitamin D changes, accounted for inter-individual variability and effect modification by supplementation mode. Vitamin D repletion in patients who were deficient at baseline may have resulted in meaningful changes in cytokine levels, however, our study was not designed to address this question. The lack of a placebo arm with 73% of patients in the study achieving 25(OH)D levels greater than 30 μg/L may be why we did not see as impressive results and needed to do secondary analysis comparing change in cytokines. Nevertheless, this study provides novel hypothesis-generating insights at the impact of vitamin D supplementation on cytokine response post-HSCT.

In summary, this is the first study to assess the cytokine response to vitamin D supplementation during HSCT in children. Vitamin D deficiency affects the pre-transplant cytokine milieu, and how that affects clinical outcomes post-transplant is an important knowledge gap that needs to be addressed. Stoss therapy appears to differ in influence on proinflammatory cytokine responses in a sex-specific or conditioning specific manner, indicating higher doses of vitamin D maybe needed to sufficiently achieve its immunomodulatory benefits. However, the small sample size in this study precludes making strong conclusions. A larger clinical trial in HSCT, including analysis of other immunological parameters, that will explore the role of vitamin D supplementation in regulating cytokine responses with relevant clinical outcomes is warranted.

## Data Availability Statement

The raw data supporting the conclusions of this article will be made available by the authors, without undue reservation.

## Ethics Statement

The studies involving human participants were reviewed and approved by Phoenix Children's Hospital Institutional Review Board. Written informed consent to participate in this study was provided by the participants' legal guardian/next of kin.

## Author Contributions

AN and JB designed research. JB, AN, KB, CC, CS, DS, HM, RA, PC, BH, SB, and TM conducted research. AN, BO, and LM analyzed data. AN, AG, BH, and LM wrote the paper. AN had primary responsibility for final content. All authors read and approved the final manuscript.

## Funding

Grants from the Learners Research Fund, Phoenix Children's Hospital and Valley Research Partnership Grant (P1201707), University of Arizona, provided support for the trial.

## Conflict of Interest

The authors declare that the research was conducted in the absence of any commercial or financial relationships that could be construed as a potential conflict of interest.

## Publisher's Note

All claims expressed in this article are solely those of the authors and do not necessarily represent those of their affiliated organizations, or those of the publisher, the editors and the reviewers. Any product that may be evaluated in this article, or claim that may be made by its manufacturer, is not guaranteed or endorsed by the publisher.
