# Supplementary material for: Vitamin D Supplementation: Association With Serum Cytokines in Pediatric Hematopoietic Stem Cell Transplantation
Source: Front Pediatr. 2022 Jul 13;10:913586. doi: 10.3389/fped.2022.913586 (PMC9326107; doi:10.3389/fped.2022.913586)
Supplement: Supplementary file 2 [file Table_2.docx]

**Supplemental Table 2**. Association of cytokines and vitamin D levels at 30 days.

| **Cytokine** | **Vitamin D 30 days post HSCT**  Mean (SD) | | | **P value^1^** | **Mean Difference**  **(95% CI)** |
| --- | --- | --- | --- | --- | --- |
|  | **All Subjects**  (N= 38) | **< 30 µg/l**  (N = 10) | **≥ 30 µg/l**  (N = 28) |  |  |
| ***Growth factors*** | | | | | |
| EGF | 3.60 (3.72) | 3.22 (1.42) | 3.73 (4.26) | 0.24 | -0.51  (-2.38, 1.36) |
| FGF | 3.34 (0.41 | 3.30 (0.15) | 3.36 (0.48) | 0.63 | -0.06  (-0.27, 0.14) |
| GCSF | 3.28 (1.70 | 3.95 (2.24) | 3.04 (1.43) | 0.41 | 0.91  (-0.75, 2.56) |
| HGF | 12.58 (37.7) | 5.24 (16.6) | 15.2 (42.7) | 0.71 | -9.96  (-29.5, 9.58) |
| ***Proinflammatory Classic*** | | | | | |
| TNFα | 0.32 (0.06) | 0.31 (0.04) | 0.32 (0.06) | 0.75 | 0.01  (-0.05, 0.02) |
| IL1A | 0.16 (0.77) | 0.01 (0.02) | 0.22 (0.89) | 0.17 | -0.21  (-0.55, 0.14) |
| IL1β | 3.73 (6.43) | 2.08 (1.26) | 4.32 (7.40) | 0.36 | -2.24  (-5.21, 0.72) |
| IL1RA | 1.17 (0.98) | 1.04 (0.74) | 1.22 (1.06) | 0.89 | -0.21  (-0.55, 0.14) |
| ***Type 1*** | | | | | |
| IL12 | 4.61 (8.25) | 1.39 (1.87) | 5.77 (9.32) | 0.10 | -4.38  (-8.17, -0.59) |
| IL2 | 0.08 (0.14) | 0.05 (0.06) | 0.09 (0.15) | 0.65 | -0.04  (-0.11, 0.03) |
| IL2R | 26.8 (88.9) | 59.9 (165) | 15.0 (34.4) | 0.66 | 44.9  (-73.7, 163) |
| ***Type 2*** | | | | | |
| IL3 | 0.51 (0.29) | 0.43 (0.21) | 0.54 (0.32) | 0.43 | -0.11  (-0.29, 0.07) |
| IL4 | 0.54 (1.70) | 0.23 (0.09) | 0.66 (1.97) | 0.67 | -0.43  (-1.20, 0.34) |
| IL6 | 8.96 (49.5) | 2.66 (7.17) | 11.2 (57.6) | 0.44 | -8.54  (-31.7, 14.2) |
| ***Chemokines*** |  |  |  |  |  |
| IL8 | 3.09 (10.2) | 7.39 (19.8) | 1.56 (1.80) | 0.96 | 5.83  (-8.37, 20.0) |
| IP10 | 5.98 (4.46) | 5.16 (4.42) | 6.28 (4.52) | 0.64 | -1.11  (-4.58, 2.35) |
| MCP1 | 78.4 (253) | 143 (411) | 55.4 (171) | 0.78 | 87.5  (-211, 386) |
| MIG | 3.03 (6.33) | 1.96 (2.98) | 3.41 (7.17) | 0.59 | -1.45  (-4.80, 1.90) |
| MIP1β | 2.24 (3.65) | 1.53 (1.86) | 2.50 (4.10) | 0.34 | -0.97  (-2.95, 1.01) |
| RANTES | 138 (33.8) | 149 (26.0) | 133.6 (35.7) | 0.15 | 15.6  (-6.45, 37.7) |
| EOTAXIN | 8.91 (6.52) | 8.62 (3.80) | 9.01 (7.31) | 0.54 | -0.40  (-4.13, 3.34) |

CI = confidence interval

^1^ P-value from the Wilcoxon rank sum test
