# Supplementary material for: Vitamin D Supplementation: Association With Serum Cytokines in Pediatric Hematopoietic Stem Cell Transplantation
Source: Front Pediatr. 2022 Jul 13;10:913586. doi: 10.3389/fped.2022.913586 (PMC9326107; doi:10.3389/fped.2022.913586)
Supplement: Supplementary file 3 [file Table_3.docx]

**Supplemental Table 3**. Association of cytokines and vitamin D levels at 30 days, when compared by type of vitamin D supplementation.

| **Cytokine** | **Baseline**  Mean (SD) | | **P value^1^** | **30 days post HSCT**  Mean (SD) | | **P value** |
| --- | --- | --- | --- | --- | --- | --- |
|  | **Stoss**  (N=19) | **Standard Care**  (N=22) |  | **Stoss**  (N=18) | **Standard Care**  (N=20) |  |
| ***Growth factors*** | | | | | |  |
| EGF | 3.75 (3.03) | 3.78 (1.85) | 0.65 | 3.62 (3.53) | 3.57 (3.97) | 0.87 |
| FGF | 3.30 (0.19) | 3.32 (0.14 | 0.89 | 3.29 (0.34) | 3.39 (0.48) | 0.78 |
| GCSF | 3.36 (1.22) | 3.81 (2.13 | 0.95 | 3.00 (1.35) | 3.53 (1.95) | 0.60 |
| HGF | 22.8 (74) | 0.00 (0.00) | 0.13 | 11.0 (35.6) | 14.0 (40.3) | 0.75 |
| ***Proinflammatory Classic*** | | | | | |  |
| TNFα | 0.33 (0.08) | 0.32 (0.05) | 0.44 | 0.31 (0.05) | 0.32 (0.06) | 0.70 |
| IL1A | 0.19 (0.52) | 0.07 (0.12 | 0.30 | 0.29 (1.11) | 0.04 (0.09) | 0.44 |
| IL1β | 3.69 (3.57) | 2.74 (1.53 | 0.82 | 2.64 (2.35) | 4.72 (8.56) | 0.62 |
| IL1RA | 1.44 (1.61) | 1.74 (2.40 | 0.73 | 1.08 (0.97) | 1.26 (1.00) | 0.49 |
| ***Type 1*** | | | | | |  |
| IL12 | 4.53 (6.21) | 3.80 (3.91 | 0.61 | 6.52 (11.2) | 2.89 (3.68) | 0.58 |
| IL2 | 0.07 (0.10) | 0.06 (0.06 | 0.56 | 0.11 (0.18) | 0.05 (0.06 | 0.55 |
| IL2R | 1.34 (2.70) | 10.8 (47) | 0.73 | 9.95 (14.9) | 42.0 (121) | 0.83 |
| ***Type 2*** | | | | | |  |
| IL3 | 0.58 (0.40 | 0.48 (0.21) | 0.57 | 0.57 (0.33) | 0.46 (0.25) | 0.24 |
| IL4 | 0.30 (0.20 | 0.26 (0.14) | 0.63 | 0.83 (2.46) | 0.28 (0.19) | 0.85 |
| IL6 | 0.34 (0.92 | 0.34 (0.86) | 0.93 | 17.2 (71.8) | 1.57 (5.09) | 0.41 |
| ***Chemokines*** |  |  |  |  |  |  |
| IL8 | 4.38 (8.55 | 2.83 (9.62) | 0.18 | 1.08 (0.97) | 4.90 (14.0) | 0.44 |
| IP10 | 4.27 (4.70) | 3.24 (3.55) | 0.47 | 6.25 (5.45) | 5.74 (3.47) | 1.00 |
| MCP1 | 103 (317) | 102 (298) | 0.77 | 66.4 (210) | 89.3 (291) | 0.41 |
| MIG | 3.18 (6.99) | 14.2 (65) | 0.45 | 3.92 (8.84) | 2.23 (2.60) | 0.92 |
| MIP1β | 3.12 (3.23) | 4.65 (6.84) | 0.50 | 1.27 (1.18) | 3.12 (4.79) | 0.06 |
| RANTES | 145 (31.13) | 130 (41) | 0.34 | 127 (38.6) | 147.8 (25.9) | 0.09 |
| EOTAXIN | 5.99 (3.84) | 7.49 (4.07) | 0.22 | 8.95 (7.67) | 8.88 (5.49) | 0.59 |
